# Single-step extraction of small-diameter single-walled carbon nanotubes in the presence of riboflavin

**DOI:** 10.3762/bjnano.13.130

**Published:** 2022-12-22

**Authors:** Polina M Kalachikova, Anastasia E Goldt, Eldar M Khabushev, Timofei V Eremin, Timofei S Zatsepin, Elena D Obraztsova, Konstantin V Larionov, Liubov Yu Antipina, Pavel B Sorokin, Albert G Nasibulin

**Affiliations:** 1 Skolkovo Institute of Science and Technology, 3 Nobel Street, Moscow, 121205, Russia; 2 Aalto University School of Chemical Engineering, Kemistintie 1, 02015, Espoo, Finland; 3 A. M. Prokhorov General Physics Institute of RAS, 38 Vavilov Street, Moscow, 119991, Russia; 4 Moscow Institute of Physics and Technology, 9 Institutskiy per., Dolgoprudny, 141701, Russia; 5 Department of Chemistry, M.V.Lomonosov Moscow State University, Moscow, 119992, Russia; 6 National University of Science and Technology "MISiS", 4 Leninsky prospect, Moscow, 119049, Russia; 7 Emanuel Institute of Biochemical Physics, Russian Academy of Sciences, 4 Kosygina st., Moscow, 119334, Russia

**Keywords:** carbon nanotubes, photoluminescence spectroscopy, riboflavin, size exclusive gel chromatography, SWCNT extraction

## Abstract

We propose a novel approach to disperse and extract small-diameter single-walled carbon nanotubes (SWCNTs) using an aqueous solution of riboflavin and Sephacryl gel. The extraction of small-diameter semiconducting SWCNTs was observed, regardless of the initial diameter distribution of the SWCNTs. Dispersion of SWCNTs occurs due to the adsorption of π-conjugated isoalloxazine moieties on the surface of small-diameter nanotubes and interactions between hydroxy groups of ribityl chains with water. During the SWCNT extraction, specific adsorption of riboflavin to SWCNTs leads to the minimization of interactions between the SWCNTs and gel media. Our experimental findings are supported by ab initio calculations demonstrating the impact of the riboflavin wrapping pattern around the SWCNTs on their interaction with the allyl dextran gel.

## Introduction

The unique physical and chemical properties of single-walled carbon nanotubes (SWCNTs) promise multiple high-end applications varying from biomedicine to photonics and electronics [1–3]. Rapid technology development and growing demand for SWCNTs led to the upscaling of nanotube synthesis from laboratory reactors to cutting-edge manufacturing all over the world. Usually, raw SWCNTs consist of highly bundled structures due to strong van der Waals interactions between nanotubes, which alter and deteriorate their outstanding intrinsic properties [3–5]. Moreover, as-synthesized SWCNTs typically possess a wide range of diameters and chiralities, leading to an inhomogeneous distribution of optical and electronic properties [5]. Nevertheless, most SWCNT applications, including thin-film electronics, require individual constituent parts. Biomedical applications apply an additional constraint on the diameter of nanotubes. Small-diameter SWCNTs display intrinsic photoluminescence in the spectral range of 900–1100 nm within the biological transparency window, making them ideal candidates for single-molecule biosensors or biomedical imaging agents [6–8].

Despite significant progress toward the synthesis of monochiral and chirality-enriched carbon nanotubes, further improvements are of unmet need even at the laboratory scale [3,8]. As SWCNT synthesis yields a distribution of bundled (*n,m*) types, solubilizing and isolating specific carbon nanotube geometries remain one of the paramount technological challenges for their potential applications [2,5,8–10].

The noncovalent functionalization of carbon nanotubes promotes their individualization due to hydrophobic interactions between nanotubes and surfactant molecules that also facilitate chirality separation. Conventional surfactants such as sodium dodecyl sulfate (SDS) and sodium deoxycholate (DOC), as well as polyethylene glycol-based compositions, are used to obtain high-quality dispersions of individual SWCNTs [3–5,10]. Although given surfactants show exemplary performance in both individualization and chirality separation of carbon nanotubes, excessive surfactant concentrations are usually required for their complete individualization [2,8,11]. Such excess subsequently introduces an additional step of surfactant removal to recover the SWCNTs in a pristine state and discard toxic residual surfactants, which would otherwise limit biological applications. Biopolymers such as DNA and RNA have been widely proven to disperse SWCNTs. Nucleic acids even exhibit sequence-dependent wrapping around nanotubes with different chiralities [12–15]. The remarkable biocompatibility of nucleic acids can support biomedical applications of such dispersions. Unfortunately, an extensive ultrasonic treatment required to obtain a dispersion of individual nanotubes might destroy fragile nucleic acid molecules so that their applications are somewhat inhibited.

Flavin compounds are another class of biomolecules that can be potentially utilized as a surfactant for dispersing SWCNTs. Having relatively low solubility in water, flavins are generally innocuous for living cells. Riboflavin (also known as vitamin B_2_) is a precursor of such coenzymes as flavin mononucleotide phosphate and flavin adenine dinucleotide [16]. The presence of π-conjugated isoalloxazine in riboflavin drives binding to SWCNTs, and the hydrophilic ribityl chain allows for the solubilization of SWCNTs in aqueous media. The planar isoalloxazine structure, accompanied by intermolecular interactions with nanotubes through π–π interaction and with neighboring isoalloxazine groups via hydrogen bonding, promotes the ordered assembly of riboflavin molecules on the surface of SWCNTs [9,17]. Papadimitrakopoulos et al. described the helical wrapping of flavin mononucleotide through π–π interaction between the isoalloxazine rings and a sidewall of SWCNTs [17–18]. Flavin derivatives compounds are known to extract specific (*n*,*m*) SWCNTs from dispersions in organic solvents [19–20]. Moreover, flavin mononucleotide phosphate could be used as a stabilizer for graphene aqueous dispersions [21]. Although proven to solubilize SWCNTs, no data on chirality separation of SWCNTs using pure aqueous riboflavin is available to date.

Here, we report the dispersion of single-walled carbon nanotubes by aqueous riboflavin solution to extract a small-diameter fraction of SWCNTs from polydisperse samples by highly-efficient single-step gel filtration. We found that the riboflavin molecules are selectively adsorbed on small-diameter semiconducting SWCNTs facilitating specific hydrophobic interactions between the nanotubes and gel extraction media consistent with first-principles calculations.

## Results and Discussion

### Riboflavin as stabilizing agent for aqueous SWCNT dispersions

**Figure 1:**
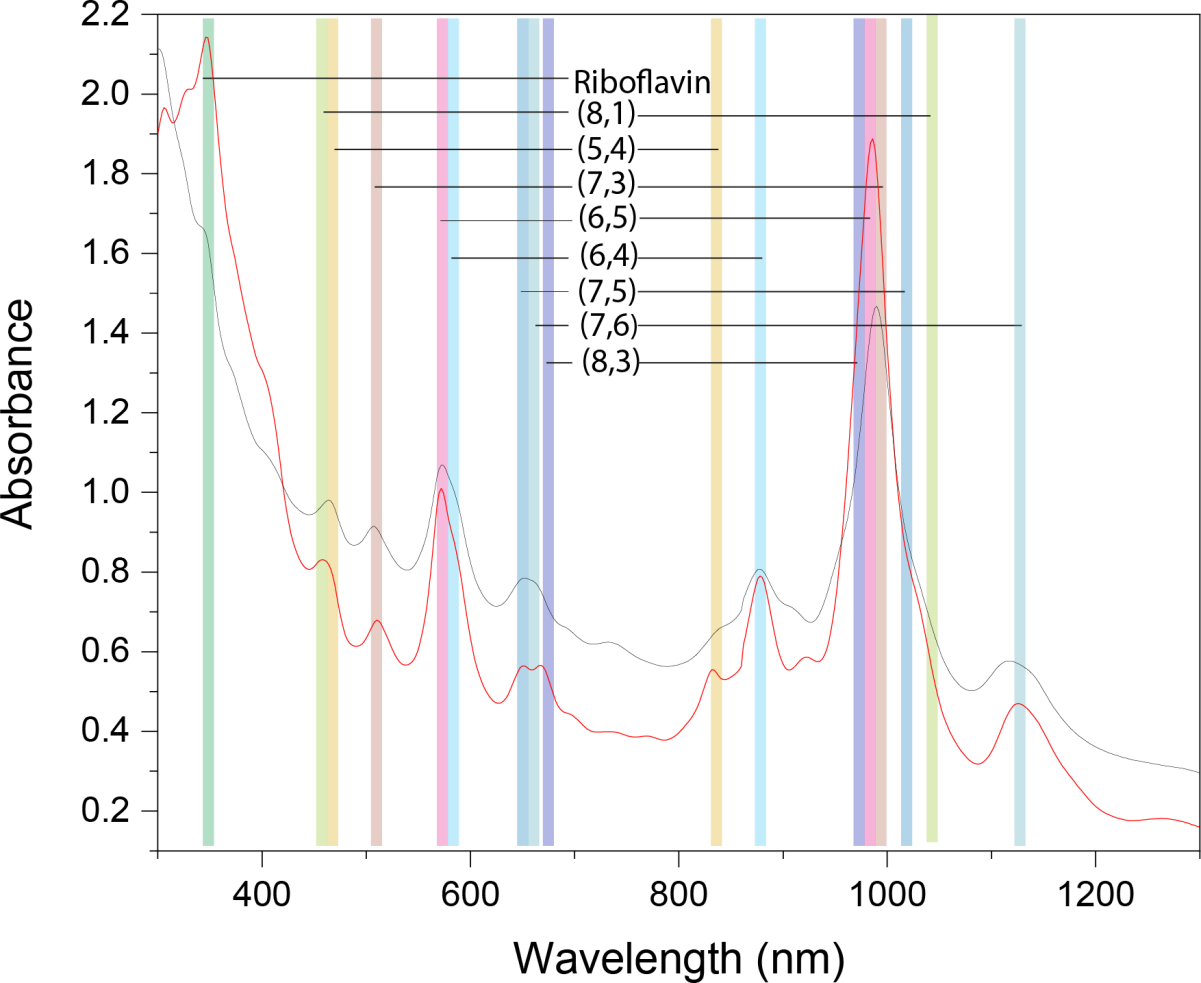
Absorbance spectra of CoMoCat SWCNT dispersions in riboflavin (red) and SDS (black).

The UV–vis–NIR spectrum of CoMoCat SWCNT/riboflavin dispersion obtained by mild sonication reveals the distinctive S_11_ and S_22_ transitions of (6,4)- and (6,5)-SWCNTs, as well as other resolved optical transitions of nanotubes (Figure 1a). Notably, Van Hove transitions of SWCNTs in riboflavin are as prominent as in aqueous SDS solution and remain unaltered even after three months, signifying a high quality of SWCNT dispersion. Such stabilization of individual nanotubes is explained by the interdigitation of noncovalently bonded riboflavin molecules with individualized nanotubes.

### Selective extraction of small-diameter semiconducting SWCNTs

Riboflavin demonstrates high adhesion to the SWCNT surface; thus, it can be utilized as both surfactant and eluent to individualize SWCNTs and precisely extract those that feature a denser riboflavin coating from samples with broad diameter distribution. The single-step extraction procedure is based on the gel filtration of SWCNTs dispersions, where manifold interactions between SWCNTs, surfactant molecules, and the polysaccharide gel govern the separation of nanotubes [22]. The classical route of gel filtration utilizes SDS or its combinations with cholic acid salts as eluting agents [8,12,14,22–23]. Metallic SWCNTs exhibit a denser packing of surfactants on their surface, and their interactions with the gel media are minimal, so they are usually collected in the first fraction. Meanwhile, semiconducting SWCNTs are entrapped in the gel and could be gradually eluted with surfactant eluent [22–23]. With riboflavin, it is possible to perform the extraction of small-diameter near-armchair SWCNTs in a single step due to the absence of eluent and the differences in the density of riboflavin coating imposed by the electronic structure of nanotubes.

**Figure 2:**
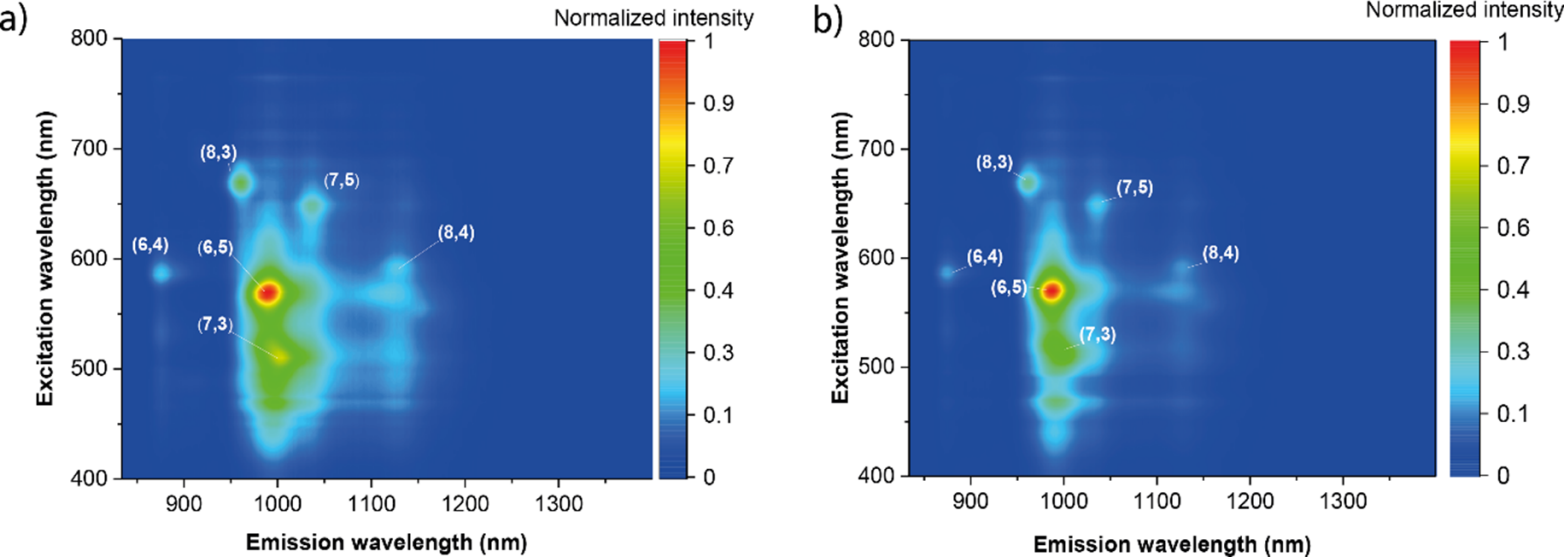
PL maps of CoMoCat SWCNTs in riboflavin dispersion (a) before and (b) after single-step extraction.

To gain insights into the correlation between SWCNT diameter and the adsorption of riboflavin, we performed single-step chirality enrichment of SWCNT dispersions with various diameter distributions: CoMoCat SWCNTs with a mean diameter 0.81 nm [24] and Tuball nanotubes with an average diameter of 1.5 nm. The set of chiralities present in CoMoCat demonstrates a high affinity towards riboflavin, leading to a high riboflavin density on the SWCNT surface. As a result, we do not observe significant changes in UV–vis–NIR spectra or photoluminescence of dispersions before and after single-step extraction (Figure 2). Since riboflavin excellently wraps small-diameter semiconducting SWCNTs, their concentration in the CoMoCat riboflavin sample was high enough to cause the reabsorption of photoluminescence (Figure S1, Supporting Information File 1).

**Figure 3:**
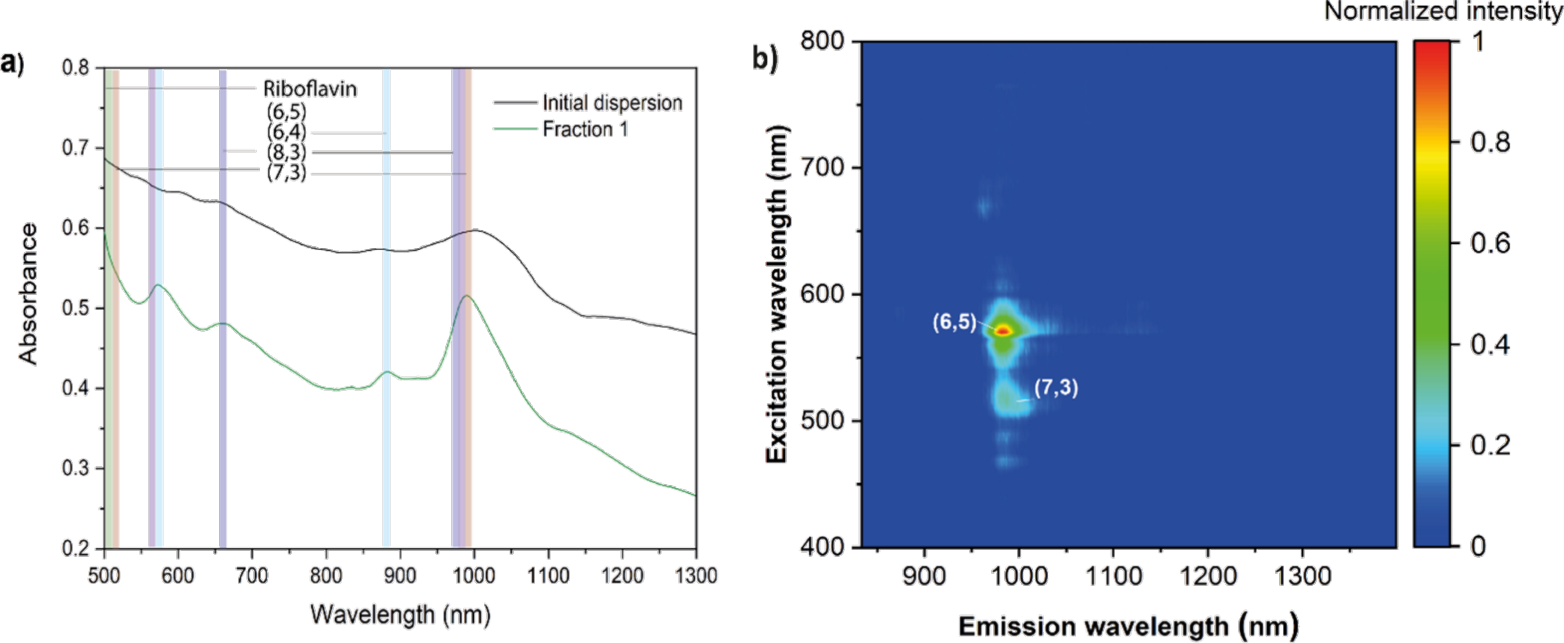
(a) Optical spectra of Tuball SWCNTs in riboflavin dispersions: initial dispersion (black) and first eluted fraction immediately after extraction (green); (b) PL map of Tuball SWCNTs in riboflavin dispersion after single-step gel extraction.

In contrast, the optical spectrum of Tuball SWCNTs after extraction features a distinctive S_11_ transition of SWCNTs at 990 nm, corresponding to (6,5)-chirality (Figure 3), unresolved before separation. The obtained absorption bands are slightly redshifted compared to the standard wavelengths of (6,5)-SWCNT absorption, which could be attributed to solvatochromism induced by noncovalent interactions between nanotubes and the aromatic isoalloxazine moiety of riboflavin in aqueous solution. The PL map of the first eluted fraction of Tuball in riboflavin-separated dispersion (Figure 3b and Figure S2, Supporting Information File 1) reveals almost single (6,5)-chiral SWCNTs with traces of (7,3)-SWCNTs, which is in agreement with the UV–vis–NIR spectrum.

### Ab initio calculations of riboflavin binding to SWCNTs

Papadimitrakopolous et al. showed that flavin mononucleotide selectively binds to (8,6)-nanotubes resulting in the formation of coaxial helices [18]. Our observations suggest that a similar wrapping pattern takes place due to the interaction between isoalloxazine rings on the surface of nanotubes, which promotes selective extraction of (6,5)-nanotubes.

**Figure 4:**
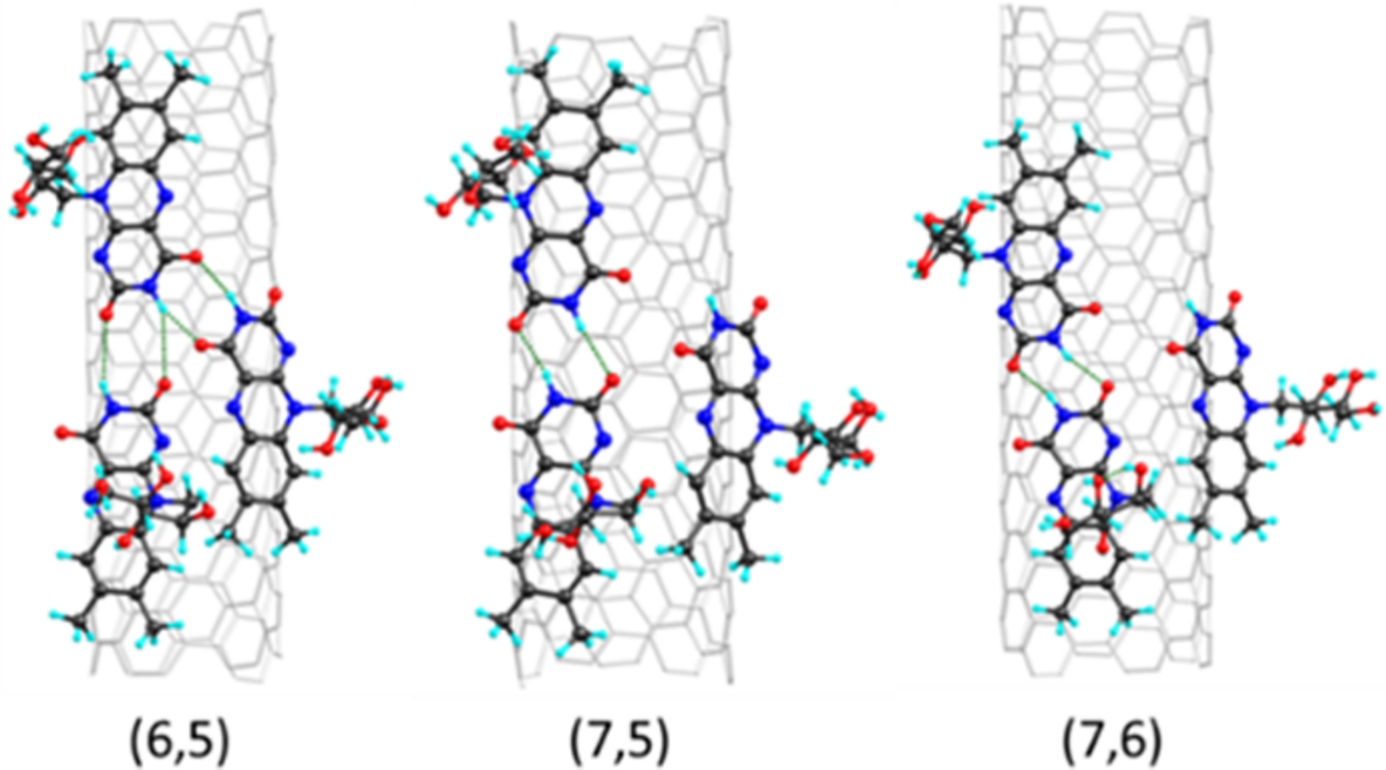
Riboflavin assembly around (6,5)-, (7,5)-, and (7,6)-chiral SWCNTs. SWCNTs are depicted by light grey sticks. Carbon, nitrogen, oxygen, and hydrogen atoms are shown with black, blue, red, and cyan colors, respectively.

Riboflavin wrapping around SWCNTs of different chiralities was simulated according to Sharifi et al. [17], where the optimal lumiflavin packing density on the SWCNT surface was derived from nanotube chirality and found to be closely connected with the strength of H-bonding between adjusted molecules (Figure 4). Such packing comes from a continuous helical wrapping of riboflavin’s isoalloxazine fragments that emerges from interfragmentary binding and concentric π–π stacking with the surface of the underlying nanotube. According to this theoretical model and experimental data, we have studied (6,5)-, (7,5)-, and (7,6)-SWCNTs, which feature different riboflavin bonding patterns as discussed previously [17]. Despite the extraction of (8,3)-SWCNTs was experimentally observed, such nanotubes feature wrapping patterns identical to those of (6,5)-SWCNTs and thus were not addressed separately.

### Calculations of interactions of riboflavin-wrapped SWCNTs with Sephacryl gel

**Figure 5:**
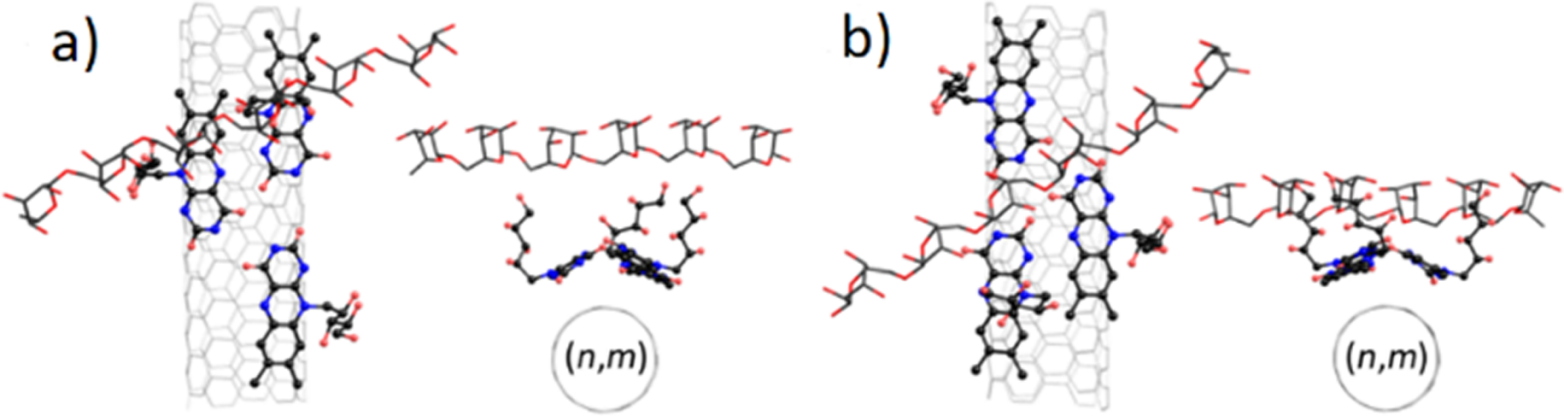
Top and front views of SWCNT–riboflavin–dextran aggregates: (a) over two ribityl chains and (b) at the edge of adjacent riboflavin molecules. SWCNTs are depicted by light grey sticks. Carbon, nitrogen, and oxygen atoms are shown with black, blue, and red colors, respectively. Dextran molecules are depicted with sticks; hydrogen atoms are hidden for simplicity.

Sephacryl gel is an allyl dextran copolymer, and its dextran links act as adsorption sites for SWCNTs in gel chromatography separation [25]. As SWCNTs are wrapped by riboflavin, there are two possible sites of SWCNT–dextran interaction, namely over two ribityl side chains (Figure 5a) and on the edge of adjacent riboflavin molecules (Figure 5b). As the riboflavin–dextran complex was relaxed, the binding energy between the riboflavin assembly and the dextran molecule was calculated.

It was found that in the case of dextran in the top position (Figure 5a), weak van der Waals interactions do not depend significantly on the SWCNT chirality and are in the range from −10 to −8.7 meV/atom (Table 1). This difference could be caused by fluctuations of ribityl chains that are not affected by the helical packing density of isoalloxazine groups. At the same time, if a dextran molecule is located in the center of the riboflavin assembly (Figure 5b), different bonding patterns are observed depending on the mutual orientation of the adjacent isoalloxazine moieties.

**Table 1:** The binding energy between riboflavin assembly and a dextran molecule for top and center deposition sites.

SWCNT (*n*,*m*)	*E*_bind_ (top), meV/atom	*E*_bind_ (center), meV/atom

(6,5)	−10.0	−11.7
(7,5)	−8.7	−18.6
(7,6)	−8.8	−22.4

The strength of bonding between neighboring riboflavin molecules is proportional to the type of riboflavin helix around a particular SWCNT. The yield of extraction, as evidenced by the decrease in photoluminescence intensity, strongly correlates with the diameter of the nanotubes and the density of riboflavin molecules on the surface of the nanotubes. (6,5)-SWCNTs exhibit the highest density of riboflavin packing, which diminishes the binding between riboflavin and allyl dextran deposited between adjacent molecules of riboflavin due to formation of two additional H-bonds with the isoalloxazine groups of riboflavin molecules (Figure 4). As a result, (6,5)-nanotubes display minimal interactions with the Sephacryl gel and are easily eluted. (8,3)-SWCNTs, although they have a diameter close to that of (6,5)-SWCNTs and have similar riboflavin wrapping patterns, do not display significant extraction. In contrast, (7,6)-SWCNTs feature a lower riboflavin packing density. They have lower energy of adsorption to Sephacryl, so their elution is hindered.

### Application trends of riboflavin-stabilized SWCNTs

Small-diameter SWCNT–riboflavin conjugates represent a promising class of nanomaterials for cancer treatment and targeted riboflavin delivery [26–28]. It has been shown that riboflavin carrier protein is highly overexpressed in several cancer tissues such as melanoma, luminal 45 A breast cancer, and squamous cell carcinoma. Riboflavin-covered SWCNTs have immense potential in detecting tumors since riboflavin is selectively attached to the riboflavin carrier protein in the tumor cells while the photoluminescence increased by SWCNTs allows for high-resolution imaging of specific tissues [29]. Notably, single-step extraction of carbon nanotubes in an aqueous media without surfactants or organic additives can significantly shorten the path from industrial or laboratory reactors to in vitro and in vivo biomedical research and further.

## Conclusion

We propose a novel preparative approach to enrich SWCNTs with a small-diameter fraction in aqueous dispersions by a single-step extraction procedure. The adsorption of riboflavin molecules governs the separation effect onto SWCNTs, which determines their interaction with adsorption sites of Sephacryl, and consequently, chirality separation. (6,5)-SWCNTs wrapped by riboflavin exhibit the densest riboflavin packing, which minimizes the binding between riboflavin and Sephacryl in various spatial geometries. Remarkably, this approach is proven to be effective for small-diameter SWCNTs despite the significant difference in diameter distribution of CoMoCat and Tuball SWCNTs.

According to computational results, riboflavin-wrapped (6,5)-SWCNTs have a minimal interaction energy with Sephacryl’s dextran sites of −10 meV/atom; thus, they are easily extracted in one step without the addition of any typical surfactants. Such optimization of the extraction procedure promotes biomedical applications of (6,5)-SWCNTs since final dispersions do not contain surfactants or organic solvents incompatible with living systems.

## Experimental

### Materials

CoMoCat (SG65i, ≥95% semiconducting SWCNTs) and Tuball (≥80% SWCNTs) SWCNTs were received from Sigma-Aldrich and OCSiAl, respectively, and used without further purification. Riboflavin (98%) was purchased from Alfa Aesar and used as received. Sephacryl S-200 High Resolution was supplied by Sigma-Aldrich.

### Preparation of dispersion of riboflavin-wrapped SWCNTs

40 mg of SWCNT powder was added to a 2 mM riboflavin solution in deionized water and stored for 14 days at room temperature in a dry chamber without exposure to direct sunlight. The final concentration of SWCNTs in dispersion was 1 mg/mL. Then, the dispersions were processed with ultrasonic treatment using Branson 450 digital sonifier at 90 W for 2 h in an ice bath to obtain dark green dispersions. Notably, dispersions were obtained without additional centrifugation step.

A riboflavin concentration of 2 mM corresponds to a 1:1 mass ratio between dry SWCNTs and riboflavin. At higher riboflavin concentrations, it precipitates due to its low solubility in water, which leads to poor solubilization of SWCNT bundles. Lower riboflavin concentrations did not affect the dispersion process of nanotubes in any way. To analyze the dispersing ability of riboflavin, the same amount of SWCNTs was dispersed in 1% w/w SDS aqueous solution by the same ultrasonication protocol.

### Single-step gel extraction protocol

A single-step gel extraction procedure was developed based on the size-exclusive gel chromatography method published elsewhere [22]. Medical syringes (20 mL, 10 cm in length, and 1.5 cm in inner diameter) were used as the separation columns. Allyl dextran Sephacryl S-200 gel was used as the separation medium. Columns were prepared by filling 5 mL of Sephacryl gel beads with subsequent equilibration with deionized water. The column was loaded with SWCNT dispersion in riboflavin, and target SWCNTs were eluted in the dead volume as the first fraction.

### Optical characterization

UV–vis–NIR spectroscopy measurements were performed using a Perkin-Elmer Lambda 1050 spectrophotometer in the wavelength range of 400–1300 nm to avoid the immense absorption of water in the NIR and minimize the interference of riboflavin absorption with the E_22_ transitions of SWCNTs. Photoluminescence measurements were performed with a Horiba Jobin Yvon NanoLog-4 spectrofluorometer with a nitrogen-cooled InGaAs detector. A Spectra-physics 3900s Ti:sapphire laser was used for excitation at 725 nm. The measurements were performed with the slit bandpass set to 5 nm and an acquisition time of 30 s.

### DFT calculations

Each riboflavin molecule was represented as an isoalloxazine group together with a ribityl side chain (C_17_H_20_N_4_O_6_). The DFT within the local density approximation for the exchange–correlation functional employing norm-conserving Troullier–Martins pseudopotentials and double-zeta plus polarization basis set were used for structural relaxation as implemented in the SIESTA package [30–35]. The geometrically confined systems were treated in a supercell scheme allowing at least 20 Å empty space between them to make intermolecular interactions negligible. The geometry of the structures was optimized until residual forces became less than 0.04 eV/Å. Grimme interatomic interaction was taken into account to describe the van der Waals-type of bonding [36]. The real-space mesh cutoff was set to 175 Ry, while calculations were performed in Gamma point.

SWCNT extraction occurs in a ternary system of SWCNT, riboflavin, and the dextran sites of Sephacryl gel. Dextran fragments in Sephacryl are described as a C_36_H_62_O_3_0 molecule. (6,5)-, (7,5)-, and (7,6)-SWCNTs were considered since they represent distinct values of riboflavin helical densities discussed by Sharifi and co-workers [17]. For each (*n,m*)-SWCNT, three riboflavin molecules were deposited on its surface (Figure 4) and relaxed reproducing wrapping patterns [17]. SWCNT was excluded from ongoing modeling, while nitrogen atoms were fixed to preserve the optimized curvature of the isoalloxazine groups. A dextran fragment was deposited on the riboflavin molecules (Figure 5). Dextran–riboflavin interaction was studied in two different geometries, namely over free ribityl chains or over a hydrogen bond between adjacent isoalloxazine groups of riboflavin molecules on the SWCNT. The dextran–riboflavin binding energy was calculated as *E*_bind_ = *E*_total_ − (*E*_dextran_ + *E*_riboflavin_).

## Supporting Information

Supporting Information features additional data on the reabsorption of photons in CoMoCat/riboflavin dispersions and Tuball/riboflavin photoluminescence spectra.

File 1 Additional experimental data.
