# Supplementary material for: Single-step extraction of small-diameter single-walled carbon nanotubes in the presence of riboflavin
Source: Beilstein J Nanotechnol. 2022 Dec 22;13:1564–71. doi: 10.3762/bjnano.13.130 (PMC9795860; doi:10.3762/bjnano.13.130)
Supplement: File 1 — Additional experimental data. [file Beilstein_J_Nanotechnol-13-1564-s001.pdf]

BEILSTEIN JOURNAL OF NANOTECHNOLOGY

Supporting Information

for

Single-step extraction of small-diameter single-walled carbon nanotubes in the presence of riboflavin

Polina M. Kalachikova, Anastasia E. Goldt, Eldar M. Khabushev, Timofei V. Eremin, Timofei S. Zatsepin, Elena D. Obraztsova, Konstantin V. Larionov, Liubov Yu. Antipina, Pavel B. Sorokin and Albert G. Nasibulin

Beilstein J. Nanotechnol. **2022,** *13,* 1564–1571. doi:10.3762/bjnano.13.130

Additional experimental data

License and Terms: This is a supporting information file under the terms of the Creative Commons Attribution License (https://creativecommons.org/licenses/by/4.0). Please note that the reuse, redistribution and reproduction in particular requires that the author(s) and source are credited and that individual graphics may be subject to special legal provisions.
The license is subject to the *Beilstein Journal of Nanotechnology* terms and conditions: (https://www.beilstein-journals.org/bjnano/terms)

Reabsorption of photons in CoMoCat/riboflavin dispersions

Despite optical spectroscopy is a powerful tool for SWCNT characterization, it does not reflect the degree of individualization and separation of the nanotubes. Photoluminescence spectroscopy allows one to identify individual semiconducting SWCNTs and to qualitatively evaluate the chirality distribution of the sample.

Dispersions of CoMoCat SWCNTs in riboflavin demonstrated superior quality because the as-produced CoMoCat nanotubes have a narrow diameter distribution and are enriched with small-diameter semiconducting SWCNTs. Also, their coating by riboflavin is energetically favored. However, it can cause a misinterpretation of the photoluminescence spectroscopy data as SWCNTs can reabsorb photons emitted by other nanotubes in dispersion due to their high concentration in the dispersion [1]. The effect of reabsorption does not allow one to qualitatively asses the set of chiralities present in the dispersion.

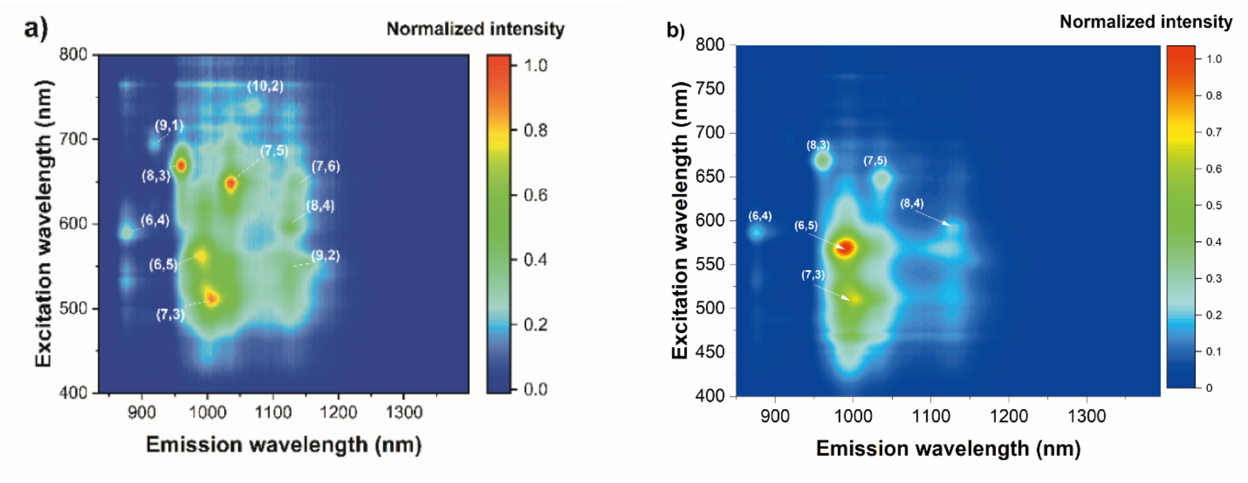

FigureS1: PL maps of the first fraction of CoMoCat in riboflavin dispersion: (a) as obtained after the chromatography column, (b) diluted five times to eliminate the reabsorption effect.

Based on Figure S1a, it can be concluded that (6,5)-SWCNTs are not the predominant SWCNTs in the dispersion, yet dilution demonstrates that it was not the case.

Nevertheless, TUBALL SWCNTs have a broader diameter distribution, and the fraction of small-diameter semiconducting nanotubes would not affect the overall spectrum regardless of the degree of their individuality.

TUBALL/riboflavin photoluminescence spectra

Figure S2 demonstrates photoluminescence spectra of TUBALL SWCNTs before and after extraction. Notably, the unseparated dispersion features one of the emission maxima at approx. 1000 nm, signifying the presence of individualized riboflavin-wrapped small-diameter SWCNTs in dispersion. However, the sample overall exhibited strong luminescence so that mapping for (*n*,*m*)-assignment would have been hindered.

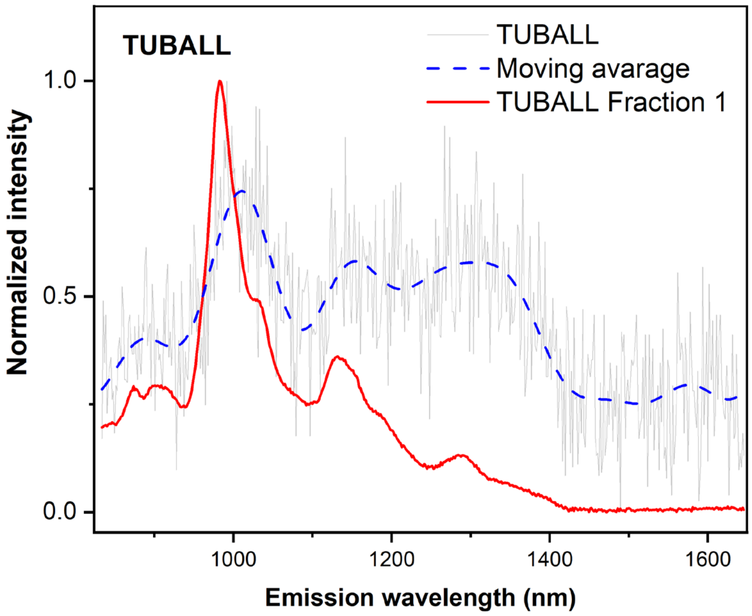

Figure S2: Photoluminescence spectra of TUBALL SWCNTs dispersed in riboflavin before (gray, blue) and after (red) single-step extraction.

References

1. Wei, X.; Tanaka, T.; Li, S.; Tsuzuki, M.; Wang, G.; Yao, Z.; Li, L.; Yomogida, Y.; Hirano, A.; Liu, H.; Kataura, H. *Nano Lett.,* **2020,** *20,* 410–417. doi:10.1021/acs.nanolett.9b04095
